# Awareness, prevalence, treatment, and control of type 2 diabetes in a semi-urban area of Nepal: Findings from a cross-sectional study conducted as a part of COBIN-D trial

**DOI:** 10.1371/journal.pone.0206491

**Published:** 2018-11-02

**Authors:** Bishal Gyawali, Martin Rune Hassan Hansen, Mia Buhl Povlsen, Dinesh Neupane, Peter Krogh Andersen, Craig Steven McLachlan, Annelli Sandbæk, Abhinav Vaidya, Per Kallestrup

**Affiliations:** 1 Department of Public Health, Aarhus University, Aarhus C, Denmark; 2 National Research Center for the Working Environment, Copenhagen, Denmark; 3 Department of Epidemiology, Welch Center for Prevention, Epidemiology, and Clinical Research Johns Hopkins Bloomberg School of Public Health, Baltimore, United States of America; 4 Rural Clinical School, University of New South Wales, Sydney, Australia; 5 Department of Community Medicine, Kathmandu Medical College and Teaching Hospital, Kathmandu, Nepal; University of Birmingham, UNITED KINGDOM

## Abstract

**Background:**

Type 2 diabetes is an escalating public health problem in Nepal. The current study aims to assess the prevalence, associated factors, awareness, treatment, and control of type 2 diabetes in a semi-urban area of Nepal.

**Methods:**

A population-based cross-sectional survey was conducted including 2,310 adults aged 25 years or above from a semi-urban area of Lekhnath Municipality of Nepal, during October 2016 to April 2017 using the World Health Organization (WHO) STEPS approach. Data on demographics, behavioral risk factors, blood pressure, anthropometric measurements (weight, height, waist and hip circumference), and fasting blood glucose were collected by face-to-face interviews during a door-to-door visit. Participants were considered to have type 2 diabetes if they had previously been diagnosed by a physician and/or were on antidiabetic medications and/or had fasting blood glucose ≥ 7.0 mmol/L. Participants were classified as being aware of their diabetes conditions if they had earlier been told that they had type 2 diabetes. Treatment of diabetes among those aware was if participants received any kind of medication treatment or counseling, and control of diabetes among those treated was defined as fasting blood glucose level was <7.0 mmol/L. Odds Ratio (OR) with 95% Confidence Interval (CI) was used to determine the strength of association.

**Results:**

The prevalence of type 2 diabetes was 11.7% (95% CI: 10.5–13.1). Among type 2 diabetes participants, 65% were aware of their disease, 94% of those who were aware received treatment, and 21% of the treated subjects had their diabetes under control. Factors significantly associated with type 2 diabetes were older age (OR = 3.2 for age group 45–54 years, OR = 3.8 for age group 55–64 years), Janajati ethnicity (OR = 1.4), abdominal obesity (OR = 2.3), being overweight or obese (OR = 1.4), and hypertension (OR = 2.0), while protective factors included being a female (OR = 0.4), medium physical activity (OR = 0.3), high physical activity (OR = 0.2), and not having family history of diabetes (OR = 0.3).

**Conclusions:**

The study revealed a high prevalence of type 2 diabetes among adults. Older age, male gender, Janajati ethnicity, abdominal obesity, overweight or obesity, hypertension, low physical activity, and family history of diabetes were associated with type 2 diabetes. Immediate public health and individual measures are warranted to reduce further burden of type 2 diabetes.

## Introduction

Global estimates of diabetes prevalence in 2014 suggested that the number of people with type 2 diabetes was 422 million and this number is projected to increase to 642 million by 2035 [1]. There is an increasing trend in the prevalence of type 2 diabetes in low-and middle-income countries (LMICs), and more than 75% adults with type 2 diabetes are now living in developing countries [1]. Furthermore, the population with prediabetes-a marker for development of type 2 diabetes-has reached approximately 318 million worldwide, equal to 6.7% of the global adult population [2]. Currently, South Asia is experiencing an increasing burden of type 2 diabetes and its complications [3]. Approximately one-fifth of all adults with type 2 diabetes in the world live in the South-East Asia Region.

Nepal is a low-income country in South Asia. While communicable diseases remain an important public health issue in Nepal, there is also a rapidly increasing burden of non-communicable diseases (NCDs), including type 2 diabetes, posing an additional burden on a resource-poor health systems. However, there is limited demographic knowledge of type 2 diabetes and its risk factors in Nepal. A 2015 systematic review and meta-analysis estimated that the prevalence of type 2 diabetes in Nepal was 8.4% (95% CI: 6.2–10.5%) [4], but the quality of the included studies were generally low, and results may not be representative for the population of Nepal as a whole.

Type 2 diabetes is mainly associated with a number of lifestyle behaviors, including daily smoking, heavy alcohol drinking, obesity, and reduced physical activity [5]. It has been revealed that behavioral risk factors are responsible for a large number of premature mortality due to cardiovascular diseases, followed by stroke [6]. More than 70% of diabetes patients die of cardiovascular events, leading to an epidemic of diabetes-related cardiovascular diseases [7]. In Nepal, risk factors for type 2 diabetes have so far rarely been investigated. However, our recent review found a number of modifiable and non-modifiable risk factors for type 2 diabetes in Nepal such as high socio-economic status, high body mass index (BMI), lack of physical activity, hypertension, alcohol and tobacco use [8]. Statistical analysis of the predictors of type 2 diabetes in Nepal has been lacking, and further research is hence needed. Moreover, diabetes awareness, treatment and control in Nepal has not received attention [8]. Our aim is to conduct a population-based study of type 2 diabetes in the semi-urban area of Lekhnath Municipality of Nepal, especially to estimate prevalence, associated factors, awareness, treatment and control of type 2 diabetes at the population level, which could help in further planning of diabetes health systems management in Nepal.

## Materials and methods

### Ethics statement

This study conformed to the Helsinki Declaration and was approved by the Nepal Health Research Council, Kathmandu, Nepal (Reg. no. 263/2016). Written informed consent was obtained from each participant before enrolling in the survey. If the participants were unable to write, then fingerprinting was used. Participants were assured verbally and in writing that all information provided would be kept strictly confidential and only used for the purpose of this study. Participants diagnosed with type 2 diabetes were referred to the nearest health facility for further treatment and follow-up.

### Study setting, design and population

This cross-sectional population-based study is a part of the Community Based Intervention for Management of Diabetes in Nepal (COBIN-D) trial (**Trial registration:** ClinicalTrial.gov: NCT03304158) [9], which was initiated in the semi-urban area of Lekhnath Municipality of Nepal (now named the Pokhara Metropolitan City due to recent restructuring of the state of Nepal according to the concept of a democratic federal system) situated 180 km west of the capital city Kathmandu. This semi-urban area has a total population of 58,816 with 14,937 households. The study was conducted from October 2016 to April 2017 among the participants recruited for the Community Based Management of Non-Communicable Diseases in Nepal (COBIN) study, the full details of which have been described by Neupane et al [10]. In brief, a systematic random sampling method was used to select a representative sample of the general population aged 25 years or above. A population framework of all eligible persons was prepared using the election voter’s list for 2007 (Lekhnath). The voter list contained information about the household. If there was more than one person from the same household eligible to participate in the study at the time of data collection, the Kish method was adopted to select the participant [11]. Selected persons who did not sign the written consent or were not able to complete the questionnaire were excluded.

### Sample size

The sample size was calculated based on an estimated prevalence of type 2 diabetes in Nepal of 9.5% [12], a 95% CI and the level of significance of 0.05 as recommended by the STEPS manual [13]. The total sample size estimate was adjusted using a design effect of 2. Using these values a sample size of 2,113 was derived which was adequate to provide results by 4 age groups (25–34 years, 35–44 years, 45–54 years, and 55–64 years) for each sex (total strata = 8). Assuming a response rate of 80%, the sample size was raised to 2,643 for this study.

### Study instruments

A culturally adapted, Nepali (local language) translated and previously validated World Health Organization (WHO) Stepwise Surveillance (STEPS) questionnaire was used [14]. The questionnaire is an instrument developed by WHO for collection of surveillance data on NCDs in resource poor settings, which includes socio-demographic information (age, gender, ethnicity, education, marrital status, occupation, income), behavioral characteristics (dietary habits, harmful alcohol use, current smoking, physical activity, hypertension, family history of diabetes), anthropometric measurements (height, weight, waist and hip circumference), blood glucose measurement and blood pressure measurements [15].

### Data collection

Data collection and training was carried out in accordance with the WHO STEPS approach recommended for NCD surveillance [16]. Prior to data collection, the questionnarie was pre-tested in a nearby non-study area. Necessary revisions were made to each questionnaire on the results of the pretest. Data were collected in face-to-face interviews by eight specifically trained field investigators with a background in health during a door-to-door visit.

### Blood glucose measurements

Fasting blood glucose for the subjects was estimated using a standardised digital glucometer, using the capillary finger prick method (fasting being defined as no caloric intake for at least eight hours). Participants were considered to have type 2 diabetes if they had previously been diagnosed by a physician and/or were on antidiabetic medications and/or had fasting blood glucose ≥ 7.0 mmol/L (126 mg/dL). Participants were classified as prediabetic if their fasting blood glucose levels were ≥ 6.1 mmol/L (110 mg/dL) and < 7.0 mmol/L (126mg/dL). The cut-off values were based on the 2006 WHO guidelines [17]. The fasting blood glucose test was conducted in the morning on a predetermined date. Participants were requested to fast overnight (including no smoking or drinking tea in the morning) and were reminded by telephone a day before the test. Fasting was confirmed verbally by the participants immediately before collecting the blood sample.

### Blood pressure measurements

Blood pressure was measured using a digital sphygmomanometer. Three readings of the systolic and diastolic blood pressure were taken with three-minute rest between each reading. In accordance with the WHO recommendation the mean systolic and diastolic blood pressure from the second and third readings were used for analysis. Participants were classified as hypertensive if their average systolic blood pressure was ≥ 140 mm Hg and/or their average diastolic blood pressure was ≥ 90 mm Hg, or if they reported being on regular anti-hypertensive therapy [18].

### Socio-demographic variables

Socio-demographic variables included in the study were age group in years (25–34, 35–44, 45–54, 55–64), gender (male, female), ethnicity (Upper caste, Janajati, Others- based on the classification by the Department of Health Services of Nepal [19], marital status (unmarried, married), education (low: up to primary schooling, medium: upto secondary and high schooling, high: college or university education), occupation (employee, housemaker, agriculture, labor, others), monthly household income (<20,000 Nepali Rupees (NPR) or <200 US Dollars (1 NPR = 0.01 US Dollar, August 2017), ≥20,000 NPR or ≥200 US Dollars), current smoking (yes, no), harmful alcohol use (yes, no), ≥5 servings of fruits and vegetables weekly (yes, no), abdominal obesity defined by waist-hip ratio (normal, high), overweight or obesity defined by BMI (yes, no), physical activity level (low, medium, high), and family history of diabetes (yes, no).

### Type 2 diabetes awareness, treatment, and control variables

Participants who reported that a physician ever told them they had type 2 diabetes were considered aware of their diabetic conditions. Participants were categorised as undergoing treatment if they received any kind of treatment such as insulin or anti-diabetic medications or counselling, and categorised as having good glycemic control if their fasting blood glucose level was lower than 7.0 mmol/L.

### Behavioral variables

Current smoking was defined as smoking at least one cigarette per day. Harmful alcohol use was determined from self-reported alcohol consumption during the last 30 days, and was defined as drinking 8 standard drinks or more in a single occasion per week among females and drinking 15 or more standard drinks in a single occasion per week among males. Pictorial cards showing different kinds of glasses and bowls most commonly used in Nepal were used to help the participants recall the amount of alcohol consumed. The amount, as identified by the respondent, was then used to estimate the number of standard drinks of alcohol use (one standard drink being defined as 10 grams of ethanol). Physical activity level was determined from questions on number of days and time spent on vigorous and/or moderate activities for work, travel and leisure activities. Using standard formula from the WHO STEPS, the number of Metabolic Equivalent of Task (MET) minutes per weeks were calculated and categorized as low (<600 MET minutes per week), moderate (> = 600 but <3000 MET minutes per week), and high physical activity (> = 3000 MET minutes per week). Participants self-reported their fruit and vegetable consumption in a typical week. One serving of vegetable was considered to be one cup of raw green leafy vegetables or 1/2 cup of other vegetables (cooked or chopped raw). One serving of fruit was considered to be one medium size piece of apple, banana or orange, 1/2 cup of chopped, canned fruit or 1/2 cup of fruit juice.

### Anthropometric measurements

Weight was measured using a digital scale, and height using a portable standard stature scales. BMI was calculated using the formula weight (kg)/(height^2^)(m^2^). A person was considered to be overweight or obese if BMI ≥24 kg/m^2^ (the cut-off levels for South Asians) [20]. Waist and hip circumferences were measured by John’s nonstretchable measuring tape. BMI was calculated using Central/abdominal obesity was defined by waist circumference ≥90 cm in males and ≥85 cm in females (undefined for pregnant women).

### Quality control

To ensure the validity and reliability of the data, strict protocols were implemented. All data enumerators were uniformly trained to conduct the face-to-face questionnaire interviews and to use the measurement instruments for five consecutive days. Completed questionnaires were validated in telephone interviews with selected participants. Repeated interviews or examinations were conducted if missing information was found. To ensure standardized measurements, all glucometers, sphygmomanometers, weighing scales and tape measures were assessed weekly by taking measurements on one person with each of the instruments. Moreover, for all participants who self-reported an earlier diagnosis of diabetes, the information was validated using their medical records.

### Data management and analysis

The completed questionnares were checked for completeness, sorted, and entered into the Epidata 3.1 software, and exported to the STATA statistical software version 14.1. Frequencies and percentages were calculated to identify the distribution of sociodemographic information. Chi-square test was conducted for comparing proportions of categorical variables. Univariate and multiple logistic regressions were performed to identify the associations between type 2 diabetes and its risks factors, and we calculated odds ratios (OR) with 95% confidence intervals (CIs). The covariates in the multivariate model were selected a priori based on literature, which will allow for better confounding adjustment. We also performed sensitivity analyses with the most significant variables, and there was no change in the significance of the variables. All statistical tests were two-tailed, and associations were considered to be statistically significant for a P<0.05. In all logistic regression models, we adusted for age, gender, ethnicity, marital status, education, occupation, monthly income, current smoking, harmful alcohol use, fruits and vegetable servings weekly, abdominal obesity, overweight/obesity, and physical activity, hypertension, and family history of diabetes.

## Results

### Socio-demographic characteristics of the study participants

The study invited 2,643 participants with a response rate of 87.4%. The total sample studied was 2,310, of which 1,574 (68%) were females and 736 (32%) were males. Table 1 shows the socio-demographic and behavioral characteristics of the study participants. The median age (±SD) of the study group was 47.37 (±9.95) years. In total, 31% of the study participants were in the 45-54-year age group. The majority of the study population had low education (53%), were from the Upper caste (54%), and were married (91%). We observed that 36% of the participants were engaged in agriculture. In total, 35% of the participants had hypertension. Of the study participants, 16% were current smokers, and around 13% consumed harmful amounts of alcohol.

**Table 1:** Socio-demographic and behavioral characteristics of the study population.

Characteristics	N = 2,310 (%)
**Age (years)**	
25–34	288 (12)
35–44	676 (29)
45–54	727 (31)
55–64	619 (27)
**Gender**	
Male	736 (32)
Female	1,574 (68)
**Ethnicity**	
Upper caste	1,254 (54)
Janajati	742 (32)
Others	314 (14)
**Marital status**	
Married	2,093 (91)
Unmarried	217 (9)
**Education**	
Low	1,215 (53)
Medium	969 (42)
High	126 (5)
**Occupation**	
Employee	462 (20)
Housemaker	757 (33)
Agriculture	838 (36)
Labor	69 (3)
Others	184 (8)
**Monthly income (NPR)**	
<20,000	817 (35)
≥20,000	1,493 (65)
**Current smoking**^a^	
Yes	365 (16)
No	1,945 (84)
**Harmful alcohol use**^b^	
Yes	307 (13)
No	2,003 (87)
**≥5 servings of fruits and vegetables weekly**^c^	
Yes	122 (5)
No	2,188 (95)
**Abdominal obesity**^d^	
Normal	474 (21)
High	1,836 (79)
**Overweight or Obese (Asian cut-off)**^e^	
Yes	1,422 (62)
No	888 (38)
**Physical activity**^f^	
Low	43 (2)
Medium	221 (10)
High	2,046 (88)
**Hypertension**^g^	
Yes	797 (35)
No	1,513 (65)
**Family history of diabetes**	
Yes	455 (20)
No	1,855 (80)

Note: *N* group size, *NPR* Nepalese Rupee

^a^Smoking at least one cigarette per day

^b^Drinking 8 standard drinks or more in a single occasion per week among females and drinking 15 or more standard drinks in a single occasion per week among males

^c^One serving of fruit was considered to be one medium size piece of apple, banana or orange, 1/2 cup of chopped, canned fruit or 1/2 cup of fruit juice

^d^Waist circumference ≥90 cm in males and ≥85 cm in females

^e^ BMI ≥24 kg/m^2^

^f^ Low (< 600 MET minutes per week), moderate (> = 600 but <3000 MET minutes per week), and high physical activity (> = 3000 MET minutes per week).

^g^Average systolic blood pressure was ≥140 mm Hg and/or average diastolic blood pressure was ≥90 mm Hg, or if reported being on regular anti-hypertensive therapy

### Prevalence of type 2 diabetes

**Fig 1:**
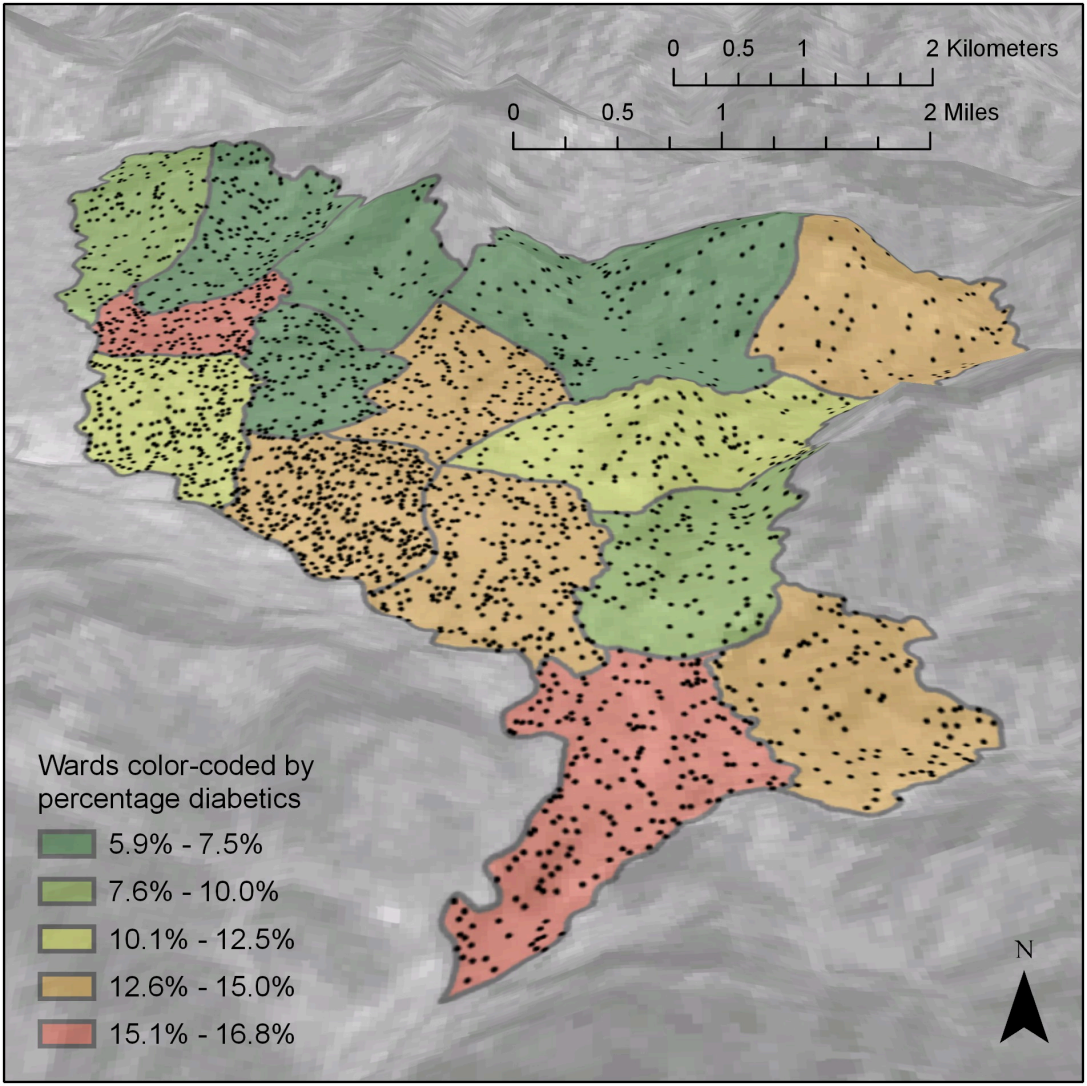
Map showing diabetes prevalence by project area. Wards in the project area are shown as individual polygons each containing a number of randomly placed dots equal to the number of participants from that ward. Map created using Esri ArcGIS Pro (Esri, Redlands, California, USA). Elevation exaggerated by a factor of 2.5. Data sources: Elevation and satellite image data available from the U.S. Geological Survey. Ward outlines from the Survey Department, National Geographic Information Infrastructure Project (Kathmandu, Nepal).

The overall prevalence of type 2 diabetes was found to be 11.7% (95% CI: 10.4–13.1), and the prevalence of prediabetes was 13.0% (95% CI: 11.8–14.5). Table 2 presents prevalence of type 2 diabetes and prediabetes stratified by age and gender. Fig 1 shows the prevalence of diabetes by administrative units (wards) of the study area.

**Table 2:** Prevalence of diabetes and prediabetes stratified by age group and gender.

Characteristics	N	Prevalence of prediabetes % (95% CI)	N	Prevalence of diabetes % (95% CI)
**Age (years)**				
25–34	19	6.5 (4.2–10.1)	11	4.1 (2.1–6.7)
35–44	81	11.9 (9.7–14.6)	49	7.2 (5.5–9.4)
45–54	109	14.9 (12.5–17.7)	98	13.4 (11.1–16.1)
55–64	93	14.9 (12.4–18.0)	113	18.2 (15.4–21.4)
**Gender**				
Male	90	12.2 (10.0–14.7)	113	15.3 (12.9–18.1)
Female	212	13.4 (11.8–15.2)	158	10.0 (8.7–11.6)
**Overall**	302	13.0 (11.7–14.5)	271	11.7 (10.2–12.8)

Diabetes is defined as individuals diagnosed by a physician and/or were on antidiabetic medications and/or those who had fasting blood glucose ≥7.0 mmol/L (≥126 mg/dL); prediabetes is defined as individuals who had fasting blood glucose levels ≥ 6.1 mmol/L (≥110 mg/dL) and <7.0 mmol/L (126mg/dL). The figures in the parentheses are expressed as percentages with 95% CIs.

### Factors associated with type 2 diabetes

Table 3 presents the results of univariate and multivariate logistic regression analysis to identify the factors associated with type 2 diabetes. On univariate analysis, the prevalence of type 2 diabetes was found to be significantly higher among those who: a) were 55–64 years (18.2%), b) were males (15.3%), c) were of Janajati ethnicity (14.9%), d) were abdominally obese (13.5%), e) were overweight or obese (14.4%), f) had low physical activity (30.0%), g) had hypertension (19.6%), or h) had a family history of diabetes (23.5%). No difference was found in prevalence by marital status, education, monthly income, current smoking, harmful alcohol use or consumption of fruits and vegetables. When these variables were entered in a multivariate logistic model, older age (both 45–54 and 55–64 years), Janajati ethnicity, abdominal obesity, overweight or obesity, and hypertension turned out to be significant risk factors (OR>1) of type 2 diabetes. Female gender, medium and high physical activity, and not having family history of diabetes were identified as significant protective factors (OR<1).

**Table 3:** Odds ratios for type 2 diabetes according to socio-demographic, behavioral and anthropometric measurement characteristics among the study population.

Characteristics	Diabetes	p-value	OR	95% CI	p-value
**Age (years)**		*p*< 0.001			
25–34	11 (3.8)		Ref		
35–44	49 (7.2)		1.7	(0.9–3.5)	0.115
45–54	98 (13.4)		3.0	(1.6–6.0)	0.001
55–64	113 (18.2)		3.6	(1.9–7.4)	*p*< 0.001
**Gender**		*p*< 0.001			
Male	113 (15.3)		Ref		
Female	158 (10.0)		0.4	(0.3–0.7)	*p*< 0.001
**Ethnicity**		0.004			
Upper caste	127 (10.1)		Ref		
Janajati	111 (14.9)		1.4	(1.0–1.9)	0.035
Others	33 (10.5)		1.2	(0.8–2.0)	0.382
**Marital status**		0.935			
Unmarried	25 (11.5)		Ref		
Married	246 (11.7)		1.0	(0.6–1.7)	0.838
**Education**		0.13			
Low	158 (13.0)		Ref		
Medium	100 (10.3)		0.7	(0.5–1.1)	0.103
High	13 (10.3)		0.6	(0.3–1.3)	0.199
**Occupation**		0.048			
Employee	46 (9.9)		Ref		
Housemaker	89 (11.7)		1.4	(0.9–2.3)	0.179
Agriculture	98 (11.6)		1.3	(0.9–2.0)	0.217
Labor	5 (7.2)		0.7	(0.3–2.1)	0.555
Others	33 (17.9)		1.2	(0.7–2.1)	0.477
**Monthly income (NPR)**		0.419			
<20,000	90 (11.0)		Ref		
> = 20,000	181 (12.1)		1.1	(0.8–1.4)	0.617
**Current smoking**		0.082			
Yes	33 (9.0)		1.5	(0.9–2.3)	0.06
No	238 (12.2)		Ref		
**Harmful alcohol use**		0.999			
Yes	36 (11.7)		0.7	(0.5–1.2)	0.215
No	235 (11.7)		Ref		
**≥5 servings of fruits and vegetables weekly**		0.121			
Yes	9 (7.3)	Ref			
No	262 (11.9)		1.7	(0.8–3.5)	0.135
**Abdominal obesity**		*p*< 0.001			
Normal	21 (4.4)		Ref		
High	250 (13.5)		2.2	(1.4–3.7)	0.001
**Overweight or obese (Asian cut off)**		*p*< 0.001			
Yes	206 (14.4)		1.4	(1.1–2.1)	0.023
No	65 (7.3)		Ref		
**Physical activity**		*p*< 0.001			
Low	13 (30.0)		Ref		
Medium	37 (16.7)		0.3	(0.2–0.8)	0.011
High	221 (10.8)		0.2	(0.1–0.5)	*p*< 0.01
**Hypertension**		*p*< 0.001			
Yes	157 (19.6)		1.9	(1.4–2.6)	*p*< 0.001
No	114 (7.5)		Ref		
**Family history of diabetes**		*p*< 0.001			
Yes	107 (23.5)		Ref		
No	164 (8.8)		0.3	(0.2–0.4)	*p*< 0.001

### Awareness, treatment, and control status of type 2 diabetes

Among all individuals identified as having type 2 diabetes, nearly two-fifths (35%) were unaware of their disease. Nearly 94% of those aware were receiving some kind of treatment such as insulin or oral anti-diabetic medications and counselling but the overall control rate was less than one quarter of those who were receiving treatment (21%) (Table 4).

**Table 4:** Awareness, treatment and control status among diabetes patients.

Demographic variables	Total diabetics N	Awareness N (%)	On treatment N (%)	Good glycemic control N (%)
Total	N = 271	N = 175 (65)	N = 164 (94)	N = 37 (21)
**Age (years)**				
25–34	11	6 (55)	5 (83)	1 (20)
35–44	49	26 (53)	23 (88)	7 (30)
45–54	98	59 (60)	56 (95)	12 (21)
55–64	113	84 (74)	80 (95)	17 (21)
**Gender**				
Male	113	77 (68)	74 (96)	14 (19)
Female	158	98 (62)	90 (92)	23 (26)

Good glycemic control was defined as fasting blood glucose <7.0 mmol/L.

## Discussion

The current study, using a representative sample from the semi-urban area of Lekhnath Municipality of Nepal, showed that 11.7% of the participants had type 2 diabetes and 13.0% of the participants had prediabetes. The diabetes prevalence was higher in the urbanized, lowland wards than in the more rural highland wards. Older age, male gender, Janajati ethnicity, abdominal obesity, overweight or obesity, hypertension, low physical activity, and family history of diabetes were associated with type 2 diabetes. Despite the high burden of type 2 diabetes, only two-thirds (65%) of participants were aware of their condition, 94% of those aware were receiving the treatment, but only about one-fifths of those on treatment had their blood glucose under control according to recommendations.

The prevalence of diabetes in our study population is consistent with a previous systematic review on diabetes in Nepal [4] that reported as pooled prevalence of 8.4%, but with prevalence estimates in individual studies ranging from 0.3% [21] to 19% [22]. Our findings are reasonably similar to diabetes prevalence estimates from studies in neighbouring South Asian countries, including India (11.1%) [23], 13.6% [24] and 18.6% [25], Bangladesh (11%) [26], Pakistan (11.1%) [27], Sri Lanka (10.3%) [28], and China (11.6%) [29]. We note that our age groups, different study populations, measurement methods, and choice of diagnostic criteria and definitions of diabetes influence prevalence estimation. Hence caution should be observed in comparisons of our findings to corresponding data from previous surveys.

In this study, age was significantly associated with type 2 diabetes and prevalence was highest among participants aged 55–64 years. A worldwide estimate for the prevalence of type 2 diabetes in 2030 predicts that in most developing countries, diabetes will be more prevalent in individuals between 45 and 64 years [30]. The importance of age as a risk factor is consistent with previous data, from various contexts [22, 26, 31].

Females were at a lower risk of type 2 diabetes compared to males in our study. This is in contrast to the findings from a meta-analysis suggesting that females were at higher risk of type 2 diabetes in Nepal (OR = 1.6; 95% CI = 1.3–1.9) [4]. Around 80% of adults in our study had abdominal obesity; and out of these 13.5% had diabetes. Similarly, more than three fifth of adults were overweight or obese (61.5%); out of which 14.5% were diabetics. Our findings underscore obesity is a risk factor for the development of type 2 diabetes as shown in previous studies [32–34]. It was reported that Asian populations are more likely to have abdominal obesity and overweight or obesity compared to their Western counterparts [35]. Similary, medium and high physical activity was associated with lower risk of type 2 diabetes in our study, corroborating findings of previous studies [36, 37]. Although there is a paucity of physical activity data in Nepal, one study revealed a high burden of physical inactivity in Nepal [38]. There is a pressing need to raise awareness on increasing physical activity and lifestyle modifications to decrease risk of type 2 diabetes.

The prevalence of type 2 diabetes in our study varied significantly with ethnicity and was highest among participants of Janajati descent. An earlier study from Nepal reported similarly a high prevalence of diabetes mellitus among Janajatis [39], and another study reported that Janajatis had the highest prevalence of overweight and obesity in Nepal [40]. Exposure to unhealthy lifestyle behaviours such as lack of physical activity and obesity as observed in our study and other studies [38] could be the contributing factors. Our study observed a positive association between hypertension and type 2 diabetes, which is consistent with a previous finding from other study conducted in a similar setting [41]. The coexistence of type 2 diabetes and hypertension might be due to sharing of common risk factors such as unhealthy lifestyle behaviors. Harmful alcohol use did not show any significant association with type 2 diabetes. This finding is consistent with studies conducted elsewhere [42, 43].

To the best of our knowledge there are no previously published studies on the awareness, treatment and control of diabetes in Nepal, and there is only limited evidence in the developing countries of South Asia. In this study, only 65% of individuals with type 2 diabetes were aware of their disease and among them, 94% were treated. Our findings were similar to findings reported in studies from Kazakhshtan [44], India [45], and Bangladesh [46]. Despite varying rates of awareness and treatment, the control rates were very low. Compared to our control rate of 21%, two different studies in China found control rates to be 27.2% and 44.2%, respectively [47, 48] and a study from Kazakhshtan found 27.7% [44]. Despite the availability of low-cost drugs for diabetes in Nepal, the overall control rate is not satisfactory. Management of diabetes is a major challenge in Nepal due to paucity of programmes to detect, manage, and prevent diabetes and its complications [8]. Nepal does not have specific guidelines regarding diabetes medication use and low medication adherence. This could be one of the barriers to proper management of diabetes. The considerably low control rate of type 2 diabetes suggests that intensive interventions and increased clinical attention should be urgently initiated among diabetics to decrease blood sugar levels.

This is one of the few studies, which surveyed prevalence and associated factors of type 2 diabetes in Nepal. The strengths of the study are random sampling of participants, interviews according to the validated STEPS questionnaire, and fasting blood glucose measurements according to the WHO recommendations. We acknowledge that the study also had a number of limitations. First, only fasting blood glucose, without other glycaemic indexes, including 2 hours post-prandial glucose or HbA1c, was used for the diagnosis of type 2 diabetes. On the other hand, this method has been reported by large cross-sectional studies conducted elsewhere [49–51]. While methods and timing of measurements may be variable, this may limit direct comparison with other published studies. It was not feasible to conduct oral glucose tolerance testing and HbA1c in the context of this large survey because of logistic and financial barriers. Consequently, we may have underestimated the true diabetes prevalence. However, all participants with known diabetes were confirmed through their medical records. The WHO considers that for epidemiological purposes, a single fasting plasma glucose estimation is acceptable [17]. Second, our study could not examine causal relationship between type 2 diabetes and demographic and behavioral factors, for which further longitudinal studies are needed. Third, the use of self-reported physical activity measures that are subjected to recall bias and over-reporting could have increased the possibility of exposure misclassification. This might have led to, for example, a higher number of individuals self reporting meeting the physical activity recommendations, thus altering the associations.

Despite the above-mentioned weaknesses, we have demonstrated a high prevalence of type 2 diabetes in Nepal that constitutes a tremendous burden to the country. The results underline the need for effective strategies for diabetes control—including prevention, surveillance and treatment. Policy makers should incorporate promotion of healthy diets and physical activity in national strategic plans to tackle NCDs, including type 2 diabetes. The study will serve as a useful tool in the planning of intervention programmes aimed at early detection of type 2 diabetes in Nepal.

## Conclusions

This study found high prevalence of type 2 diabetes, medium awareness, a high treatment rate in diagnosed cases but a suboptimal control rate. Older age, male gender, Janajati ethnicity, abdominal obesity, overweight or obesity, hypertension, low physical activity, and family history of diabetes were risk factors for type 2 diabetes. Current findings suggest a high future burden of cardiovascular diseases in Nepal. Immediate planning and implementation of public health measures and individual interventions are needed to prevent the occurance and complications of type 2 diabetes.

## Supporting information

S1 Table Minimal data set. (XLSX)
