# Correction: Awareness, prevalence, treatment, and control of type 2 diabetes in a semi-urban area of Nepal: Findings from a cross-sectional study conducted as a part of COBIN-D trial

**DOI:** 10.1371/journal.pone.0209046

**Published:** 2018-12-06

**Authors:** Bishal Gyawali, Martin Rune Hassan Hansen, Mia Buhl Povlsen, Dinesh Neupane, Peter Krogh Andersen, Craig Steven McLachlan, Annelli Sandbæk, Abhinav Vaidya, Per Kallestrup

The following information is missing from the Funding section: Publication costs were funded by the Diabetes prevention and management by lay health workers in Nepal project-Nepal Development Society (NEDS) and World Diabetes Foundation, Denmark.
